# A pilot study of intraocular use of intensive anti-inflammatory; triamcinolone acetonide to prevent proliferative vitreoretinopathy in eyes undergoing vitreoretinal surgery for open globe trauma; the adjuncts in ocular trauma (AOT) trial: study protocol for a randomised controlled trial

**DOI:** 10.1186/1745-6215-14-42

**Published:** 2013-02-13

**Authors:** Philip J Banerjee, Malcolm G Woodcock, Catey Bunce, Robert Scott, David G Charteris

**Affiliations:** 1 Moorfields Eye Hospital NHS Foundation Trust, City Road, London, EC1V 2PD, UK; 2 Royal Air force and Worcestershire Acute Hospitals NHS Trust, Charles Hastings Way, Worcester, WR5 1DD, UK; 3 Birmingham and Midland Eye Centre, Dudley Road, Birmingham, B18 7QH, UK

## Abstract

**Background:**

Eyes sustaining open globe trauma (OGT) is a group at high risk of severe visual impairment. Proliferative vitreoretinopathy (PVR) is the commonest cause of retinal redetachment in these eyes and is reported to occur in up to 45% of cases. Intensive anti-inflammatory agents have been shown to be effective at modifying experimental PVR and to be well tolerated clinically.

The Adjuncts in Ocular Trauma (AOT) Trial was designed to investigate the benefits of using intensive anti-inflammatory agents (intravitreal and sub-Tenon’s triamcinolone, oral flurbiprofen and guttae prednisolone 1.0%) perioperatively in patients undergoing vitrectomy surgery following open globe trauma.

**Methods/design:**

Patients requiring posterior vitrectomy surgery following open globe trauma will be randomised to receive either standard treatment or study treatment. Both groups will receive the standard surgical treatment appropriate for their eye condition and routine perioperative treatment and care, differing only in the addition of supplementary adjunctive agents in the treatment group. The investigated primary outcome measure is anatomical success at 6 months in the absence of internal tamponade.

**Discussion:**

This is the first randomised controlled clinical trial to investigate the use of adjunctive intensive antiinflammatory agents in patients undergoing vitrectomy following open globe trauma. It will provide evidence for the role of these adjuncts in this group of patients, as well as provide data to power a definitive study.

**EudraCT No:**

2007/005138/35

## Background

### The worldwide impact of ocular trauma

Trauma is an important cause of visual impairment and blindness worldwide and a leading cause of blindness in young adult males [1]. Currently, it is estimated that almost 1 million people in the US live with trauma-related visual impairment, and 40,000 to 60,000 individuals are diagnosed as new cases of trauma-related blindness each year [2]. Those patients with open globe injuries lose a mean 70 days of work [3]. Prevent Blindness America estimated that in 1988 approximately 90,000 disabling eye injuries occurred at the workplace, resulting in a total direct cost of $354,870,000; with indirect cost (lost wages, medical expenses, and insurance administration cost), the sum reached $709,740,000. When the cost of all non-disabling eye injuries is included, the total easily exceeds $1 billion. (Prevent Blindness America Statement on the Scope of the Eye Injury Problem, Schamburg IL, 1996). Globally it has been estimated that 1.6 million people are blind as a result of ocular trauma, with 2.3 million suffering bilateral low vision and up to 19 million with unilateral blindness or low vision [4].

In the UK it is estimated that 5,000 patients per year sustain eye injuries serious enough to require hospital admission and of these 250 will be permanently blinded in the injured eye [5]. Ocular trauma is the commonest cause of unilateral blindness in the world today and in developing countries the high incidence of ocular trauma has extensive socioeconomic costs [4].

It is clear from recent published data that although vitreoretinal surgical techniques have improved, outcomes remain unsatisfactory and that the development of proliferative vitreoretinopathy (PVR) is the leading cause of this [6-9].

### Proliferative vitreoretinopathy

Eyes sustaining penetrating or open globe trauma (OGT) are a group at high risk of severe visual impairment. Retinal detachment is common in these eyes and multiple surgical interventions are often necessary. PVR is the commonest cause of recurrent retinal detachment and visual loss in eyes with open globe trauma. It is estimated to occur in 10-45% of all OGTs [5-13].

PVR can be considered a wound-healing response in a specialised tissue. This results in the formation of fibrocellular membranes on both surfaces of the detached retina and the posterior hyaloid face. Contraction of these membranes results in retinal detachment in post-trauma eyes or in subsequent failure of surgical reattachment. Despite the improvements in vitreoretinal surgery over the past 15 years, a significant number of cases fail to achieve reattachment. The most common cause of anatomic failure in retinal detachment surgery is the development of new or recurrent proliferative vitreoretinopathy [14,15].

### Preclinical and clinical data

The cellular components of PVR peri-retinal membranes (RPE, glial, inflammatory and fibroblastic cells) proliferate and may also be contractile; they are thus targets for antiproliferative agents. There is a notable inflammatory component to the PVR process with marked blood-retinal barrier breakdown and a greater tendency to intraocular fibrin formation [16]. Macrophages and T lymphocytes have been identified in PVR membranes [17] and, although relatively small in number, they may play an important role in membrane development and contraction through growth factor production. Thus, both cellular proliferation and the intraocular inflammatory response are realistic targets for adjunctive treatments in PVR.

Steroid treatment can potentially influence both inflammatory and proliferative components of PVR. Experimental work has suggested that the corticosteroid triamcinolone acetonide can reduce the severity of PVR. In addition, it has been demonstrated that periocular corticosteroids can reduce [18,19] the severity of experimental PVR [20]. Laboratory work has also demonstrated that triamcinolone appears to have no significant retinal toxicity [21] although in vitro it may be toxic to proliferating retinal cells.

Investigation of the pathobiology of PVR has demonstrated a process of fibrocellular proliferation and contractile periretinal membrane formation, which appears to be due to migrating RPE cells [17]. Recent laboratory investigations by the senior author have demonstrated the central role of retinal glial upregulation and Müller cell process extension in the formation of PVR periretinal membranes [22,23], suggesting that adjuncts that transiently affect free-floating intraocular cells may have limited effect on the PVR process. Hence, targeting the retinal glial response may be a more effective method of modifying the clinical development or recurrence of PVR. We have subsequently tested the effect of triamcinolone on the glial response in experimental retinal detachment and found a significant reduction in Müller cell proliferation in treated animals [24]. The effect of triamcinolone on the glial response in experimental retinal detachment is evidence that clinically, in addition to their antiinflammatory effect, steroids will downregulate the intraretinal proliferative response seen in PVR.

Jonas *et al.* reported that intravitreal crystalline cortisone was well tolerated in PVR cases undergoing vitrectomy [25]. Previous small-scale, uncontrolled clinical studies of PVR have suggested that systemic prednisolone [26], infused dexamethasone [27], and intravitreal triamcinolone [28] may reduce the severity of PVR, although none of these studies were of sufficient power to provide a definitive answer. An initial pilot study by the senior author has shown that triamcinolone is well tolerated in PVR cases undergoing vitrectomy and silicone oil exchange and that a combination of adjuncts targeting the inflammatory component of the PVR process may be a potential treatment to prevent PVR [29]. A small, prospective, non-comparative clinical study by Cheema *et al.*[30] concluded that triamcinolone may have some benefit as an adjunct in non-trauma related established PVR, whereas Ahmadieh *et al.*[31] found no additional benefit in surgical outcome. Both groups concluded that further larger studies were required to definitively answer the question.

### Investigational medicinal products

Triamcinolone acetonide is a hydrophobic long-acting steroid preparation, which is increasingly being administered intraocularly to treat a variety of retinal conditions. It is cheap and widely available with a long duration of action and good safety profile. It appears to be effective in preventing intraocular proliferation and stabilising the blood-retinal barrier, making it a useful adjunctive treatment for retinal diseases resulting in refractory macular oedema. It is particularly useful in patients who have isolated ocular disease, especially unilateral, providing an antiinflammatory and antiproliferative efficacy equal to or greater than that achieved with systemic administration while avoiding the unwanted systemic side effects of steroid use. Its effects last about 3 months [32], which covers the key active developmental stage of PVR that occurs over about 6 to 8 weeks following ocular injury.

Flurbiprofen is an oral non-steroidal antiinflammatory drug most commonly used to treat musculoskeletal disorders including rheumatoid disease, osteoarthritis, and bursitis, but in ophthalmic practice it is commonly used to treat scleritis. The rationale for its use in this study stems from evidence suggesting that non-steroidal antiinflammatory medications given perioperatively may limit the degree of blood-retinal barrier breakdown and have been shown to inhibit cellular proliferation in vitro [33-35].

Prednisolone acetate 1% is an eye drop suspension used commonly in the treatment of steroid-responsive inflammatory conditions of the eye. It remains the most potent topical steroid treatment licensed for use in the UK.

Combining corticosteroids and non-steroidal antiinflammatories has been shown to have a synergistic effect in reducing blood-ocular barrier breakdown [33].

## Methods/design

This is a phase 2, single-masked, single-centre, pilot, randomised, controlled clinical trial. Forty patients will be recruited to partake in the study. Participants will be randomised into either the treatment group (additional perioperative antiinflammatory adjuncts) or control group (no additional treatment). Both groups will receive the standard surgical treatment appropriate for their eye condition and routine preoperative and postoperative treatment and care, differing only in the addition of supplementary antiinflammatory agents in the treatment group.

### Objectives

Primary:

To determine whether adjunctive use of antiinflammatory agents in eyes requiring vitrectomy following open globe trauma has an effect on anatomic reapposition of the remaining retina to the retinal pigment epithelium in the absence of an internal tamponade agent at 6 months post surgery. This pilot study aims to provide data to power a definitive future study.

Secondary:

To determine whether adjunctive use of antiinflammatory agents in eyes requiring vitrectomy following open globe trauma has an effect on:

(1) Visual acuity at 6 months (ETDRS method)

(2) Number of procedures required to achieve retinal reattachment

(3) Presence and grade of postoperative proliferative vitreoretinopathy according to the Retina Society classification on proliferative vitreoretinopathy (1991)

(4) Persistent submacular fluid found by optical coherence tomography in the presence of retinal reattachment

Further secondary objectives include providing information regarding the rate of recruitment, case mix of ocular trauma, and patient loss to follow-up rates.

### Eligibility

#### Inclusion criteria

• All patients with an open globe injury requiring vitrectomy either following open globe trauma (OGT) or as the primary procedure itself.

(OGT is classified as one of the following: The eyewall has a full-thickness injury in the form of a rupture caused by a blunt object, laceration caused by a sharp object, penetrating/perforating injury caused by a sharp object, or intraocular foreign body injury.)

In the case of bilateral injury, the worst injured eye (at the investigator’s discretion) will be included in the study.

#### Exclusion criteria

• Age < 18 or > 80 years of age

• Pre-existing glaucoma

• Previous vitrectomy surgery to the affected eye

• Pregnant or breastfeeding females

• Previous known adverse reaction to any of the IMPs

• Enrolment in other studies

• Inability to attend regular follow-up

• Unable to give informed consent

Patient recruitment will only be done when the trial has documented REC, Regulatory and Local Trust R&D approval. The study is conducted in accordance with the International Conference on Harmonisation for Good Clinical Practice, as set out in the European Union Clinical Trials Directive (2001) and associated UK Regulations (2004). The study will comply at all times with the Declaration of Helsinki (2000).

All 40 participants will be identified and recruited from outpatients and emergency referrals at Moorfields Eye Hospital. At screening, a structured interview will be conducted by research staff, including questions on coexisting ocular pathology, previous ophthalmic surgical procedures and cause of injury, to confirm that all inclusion and exclusion criteria are satisfied.

Patients are then randomised to either the treatment arm or control arm. An unstratified pre-randomised list of 40 study IDs will be held by the trials pharmacist, and following informed consent and recruitment into the trial, participants will be allocated to the lowest unused study ID. Out of hours (i.e. weekends and bank holidays), the next study ID in sequence will be kept in a sealed envelope in a secure location on site when access to the trials pharmacist is limited.

Baseline assessments will be performed within 2 weeks prior to the scheduled operation date. Data including patient demographics; site, nature and extent of ocular injury; and a full slit-lamp ophthalmic examination will be included. Where posterior chamber assessment is directly possible, retinal attachment status, presence and grade of PVR, and spectral domain OCT-guided foveal thickness and macula volume will be recorded. Where media opacity precludes a direct fundal assessment, B scan ultrasonography will be used to document retinal attachment status, and parameters regarding grade of PVR and foveal thickness and volume will be deemed unrecordable.

### Interventions

#### Treatment group

##### Preoperative Treatment

**Guttae prednisolone forte** 2 hourly for up to 1 week will replace any topical antiinflammatory agents that the patient may already be using, and all other ocular treatment will be continued

##### Perioperative Treatment

**4 mg/0.1 ml intravitreal triamcinolone acetonide** will be injected into the vitreous cavity following closure of the scleral ports at the end of the procedure.

**40 mg/1 ml triamcinolone acetonide** will be given as a posterior sub-Tenon’s injection prior to suturing the conjunctiva.

Standard subconjunctival antibiotic injection of 125 mg cefuroxime will be given.

##### Postoperative Treatment

**Guttae prednisolone forte** hourly for 1 week followed by a tapering regimen for 3–26 weeks thereafter depending on the degree of postoperative inflammation and cystoid macular oedema.

**50 mg flurbiprofen** orally bid for 1 week

Routine topical antibiotics (guttae chloramphenicol 0.5% qds for 2 weeks) and mydriatics (guttae cyclopentolate 1%, frequency and duration at surgeon’s discretion)

#### Control group

##### Preoperative

No additional treatment is given. Patients are instructed to continue with their current treatment, which may already include topical antiinflammatory agents, such as guttae dexamethasone, and topical antibiotics and mydriatics.

##### Perioperative

Standard subconjunctival medications to include 4 mg betamethasone and 125 mg cefuroxime will be given.

##### Postoperative

Routine topical antibiotics (guttae chloramphenicol 0.5% qds 2 weeks), topical steroids (guttae dexamethasone 0.1%, frequency and duration at operating surgeon’s discretion) and topical mydriatics (guttae cyclopentolate 1%, frequency and duration at operating surgeon’s discretion) will be given.

#### Masking

This is a single-masked study to participants only. Although the preoperative and postoperative regimens differ, participants are not informed to which group they have been randomised. It is not possible to mask the investigator, as the primary IMP, intravitreal triamcinolone, is clearly visible on posterior chamber assessment. The operating surgeon will be masked to the randomisation of the participant, until the end of the procedure, to avoid any bias regarding surgical management.

#### Study visits and assessment schedule

**Figure 1:**
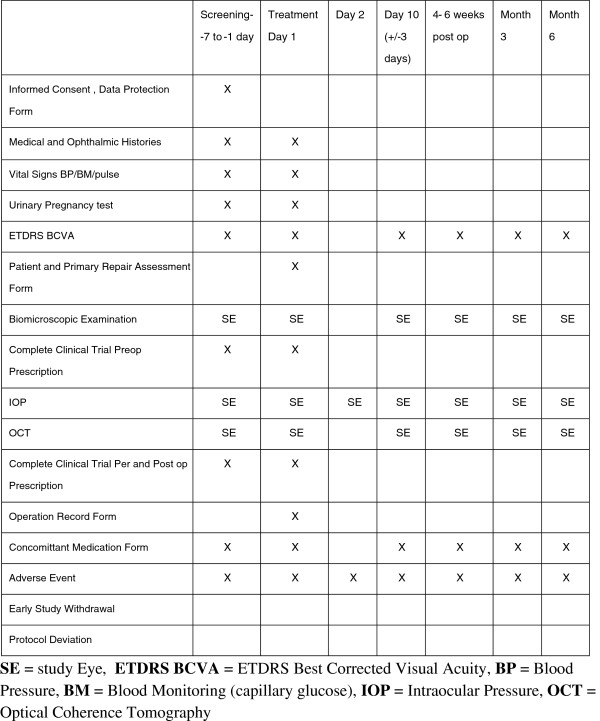
Outline of the study visit and assessment schedule.

Postoperative study visits will not differ from the routine schedule for vitreoretinal procedures at the study site, i.e. day 1, 10 days, 4–6 weeks, 3 months and 6 months. The time window allowed around these scheduled visits will be as follows: day 10 (±3 days), week 4–6 (±7 days), and months 3 and 6 (±14 days). At each scheduled postoperative study visit, a full ophthalmic assessment will be completed, to include slit-lamp biomicroscopy (± indirect binocular ophthalmoscopy when required), and parameters including ETDRS visual acuity, Goldmann applanation tonometry, anterior segment assessment and retinal attachment status will be recorded. Spectralis domain optical coherence tomography will be used to record central foveal thickness and total macula volume. (See Figure 1 for an outline of the study visit and assessment schedule.)

In patients in whom silicone oil is used as a tamponade agent, its routine removal will not be considered a reoperation, and routine subsequent follow-up will be continued until the patient can return to the study visit schedule. Other vitreoretinal interventions over the trial period will be considered reoperations and recorded as such. Postoperative visits related to reoperations, or any other attendances outside the study visit schedule, will be recorded as ‘unscheduled visits’. CRFs identical in composition to the study scheduled visit CRF will be completed and included in the data analysis on completion of the study.

Following the final study visit at 6 months, participants will be discharged back to the care of their General Practitioner. Participants requiring ongoing ophthalmic care will continue to be followed up under their admitting consultant.

### Outcome Measures

#### Primary

Successful anatomic reapposition of the remaining retina to the retinal pigment epithelium in the absence of an internal tamponade agent at 6 months post-primary vitrectomy surgery.

### Secondary

Analysis of the following secondary outcomes at 6 months post-primary vitrectomy surgery will be performed:

(1) Best corrected visual acuity using the ETDRS method

(2) Number of procedures required to achieve retinal reattachment

(3) Presence and grade of postoperative PVR [36]

(4) Persistent submacular fluid found by optical coherence tomography in the presence of retinal reattachment.

(5) Recruitment rate

(6) Case mix of ocular trauma

(7) Loss to follow-up rate

### Adverse events and safety reporting

Safety reporting will adhere to the sponsor’s standard operating procedures, and the trial team is confident that means are in place to monitor, record and report adverse events in line with the MHRA guidelines. An external Data Monitoring Committee is in place with an agreed charter to which to adhere. They will meet 6 to 12 monthly or on an *ad hoc* basis as required.

Expected adverse events will include cataract, raised intraocular pressure, hypotony, sterile hypopyon, retinal detachment, uveitis and further surgery.

Unexpected adverse events will include endophthalmitis and systemic illness.

### Trial size and duration

As this is a pilot study, a power calculation to determine sample size was not performed. A total of 40 patients was deemed a feasible number over the study period and expected to provide sufficient data to power a definitive study.

An internal audit of the incidence of open globe trauma at the study site provided data estimating an expected recruitment rate of two cases per month. This would result in the required sample size achieved in 20 months and completion of the trial in 26 months.

### Statistical analyses

The statistical analysis plan will be written in advance of the data analysis by the trial statistician and will be approved by the Trial Steering Committee and/or the Data Monitoring Committee. Data analysis will adhere to the CONSORT guidelines for randomised controlled trials. Baseline characteristics of the two groups will be compared to assess the adequacy of randomisation.

#### Primary endpoint analysis

The proportion of patients in whom anatomical retinal attachment remains at 6 months post primary vitrectomy will be reported with 95% confidence intervals.

#### Secondary endpoint analysis

Summary statistics for all secondary outcomes will be provided by treatment group, and if statistical comparisons are made, results will be reported as exploratory.

Any deviations from the statistical analysis plan will be described and justified in the final report, as appropriate.

### Trial Organisation and monitoring

#### Trial management committee

David Charteris, Chief Investigator, Moorfields Eye Hospital, London, UK

Philip Banerjee, Co-Investigator, Moorfields Eye Hospital, London, UK

Catey Bunce, Co-Investigator/Trial Statistician, Moorfields Eye Hospital, London, UK

#### Trial steering committee

Prof. Roger Hitchings, Chair, Emeritus Prof. of Glaucoma and Allied Studies University College London and Moorfields Eye Hospital, London

Hadi Zambarakji, Consultant Ophthalmologist and Clinical Trialist, Whipps Cross University Hospital NHS Trust, London

Sue Beer, Lay Person

David Charteris, Chief Investigator, Moorfields Eye Hospital, London, UK

Philip Banerjee, Co-Investigator, Moorfields Eye Hospital, London, UK

Catey Bunce, Co-Investigator/Trial Statistician, Moorfields Eye Hospital, London, UK

#### External data monitoring committee

Prof. Robert Maclaren Nuffield, Laboratory of Ophthalmology and Oxford Eye Hospital Biomedical Research Centre, University of Oxford, John Radcliffe Hospital, Oxford, UK

Timothy Jackson, Consultant Vitreoretinal Surgeon and Clinical Trialist, King’s College, London, UK

Victoria Cornelius, Senior Lecturer in Medical Statistics, King’s College, London, UK

David Broadway, Consultant Ophthalmologist, Glaucoma Specialist, Norfolk and Norwich University Hospitals NHS Foundation Trust

### Trial documentation and data collection

Case report forms will be designed and produced by the investigators, according to the sponsor’s CRF template. The final version will be approved by the sponsor. It will be the responsibility of the investigators to ensure the accuracy of all data entered onto the CRFs. A delegation log will identify all trial personnel with responsibilities for data collection and handling, including those who have access to the trial database.

Data handling will be performed adhering to the Data Protection Act 1998.

### Ethics and competent authority review

Applications to the UK main and Local Research and Ethics Committee (REC) have received favourable opinions and a Clinical Trial Authorisation has been issued by the MHRA.

### Publication policy

The results of this pilot study will be submitted for publication in peer-reviewed medical journals regardless of whether the findings are in favour of the trial interventions.

### Proposed time scale

*Trial Start -* September 2011

*Projected Trial End -* November 2013

*Trial Duration -* 24 months

*Duration of patient’s participation -* 6 months

## Discussion

This pilot randomized controlled trial investigating the use of intensive antiinflammatory agents will be the first to evaluate their role as an adjunctive agent in patients undergoing vitrectomy surgery following open globe trauma. As this group of patients is often given a poor visual prognosis, largely because of the high incidence of retinal detachment complicated by PVR, a positive outcome could potentially have significant implications in the change of management of these patients.

As this is a pilot study, it is anticipated that the results will provide sufficient valuable data to assist in powering a definitive study. As well as providing data regarding the case mix of ocular trauma at the study site, recruitment rate, and loss to follow-up rates, it aims to evaluate whether adjunctive antiinflammatory agents offer any additional benefit in this group of patients. As the sample size is small and has not been subject to a power calculation, we accept that we may limit our sensitivity to small differences between the two groups.

We also accept that the cohort of participants is likely to be quite a heterogeneous group given that open globe trauma can vary significantly in extent and severity. However, we expect the adequacy of randomization to compensate for this and shall acknowledge any unequal weighting within the groups as limitations of this pilot study. As the single-centre pilot study serves a wide geographical catchment area with a broad patient demographic representative of the UK, we do not expect the results to provide a misleading estimate of treatment effect. We would expect this to be confirmed in a definitive multicentre study.

The authors acknowledge the limitations of a single masked study, but have made every effort to reduce investigator bias by: (1) masking the operating surgeon to the treatment allocation until the end of the surgical procedure and (2) being explicit in definitions of clinical findings and adverse events and defining rigid management protocols, e.g. for IOP rise. The trial team considered an independent assessor of the primary outcome measure at 6 months, but felt that there was insufficient evidence to suggest this to be necessary and added additional cost to a pragmatic exploratory pilot study. Our projected recruitment rate is based on a retrospective audit of the incidence of relevant cases at the study site. We expect a high recruitment uptake following successful eligibility screening, due to the poor prognosis associated with current standard treatment, and are optimistic that our recruitment target be met within the projected timescale.

In summary, this is the first randomised controlled clinical trial to investigate the use of adjunctive intensive antiinflammatory agents in patients undergoing pars plana vitrectomy following open globe trauma.

## Competing interest

The authors declare that they have no competing interests.

## Authors’ contributions

PJB participated in development of the trial protocol, co-ordinated the trial’s setup at the study site, prepared the standard operating procedures, carried out study documentation, and drafted the manuscript. MGW participated in development of the trial protocol and helped to secure trial funding. CB participated in development of the trial protocol, and carried out the standard operating procedures and study documentation. RS participated in the development of the trial protocol and helped to secure trial funding. DGC conceived and designed the trial, secured trial funding, prepared the trial’s setup, prepared the standard operating procedures, carried out study documentation, and drafted the manuscript. All authors read and approved the final manuscript.
